# Quantifying the impact of delayed delivery of cardiac rehabilitation on patients’ health

**DOI:** 10.1177/2047487320912625

**Published:** 2020-03-25

**Authors:** Sebastian Hinde, Alexander Harrison, Laura Bojke, Patrick Doherty

**Affiliations:** 1 Centre for Health Economics, University of York, UK; 2 Department of Health Sciences, University of York, UK

**Keywords:** Cardiac rehabilitation, delay, uptake, completion, economic evaluation, cost effectiveness

## Abstract

**Background:**

Despite its role as an effective intervention to improve the long-term health of patients with cardiovascular disease and existence of national guidelines on timeliness, many health services still fail to offer cardiac rehabilitation in a timely manner after referral. The impact of this failure on patient health and the additional burden on healthcare providers in an English setting is quantified in this article.

**Methods:**

Two logistic regressions are conducted, using the British Heart Foundation National Audit of Cardiac Rehabilitation dataset, to estimate the impact of delayed cardiac rehabilitation initiation on the level of uptake and completion. The results of these regressions are applied to a decision model to estimate the long-term implications of these factors on patient health and National Health Service expenditure.

**Results:**

We demonstrate that the failure of 43.6% of patients in England to start cardiac rehabilitation within the recommended timeframe results in a 15.3% reduction in uptake, and 7.4% in completion. These combine to cause an average lifetime loss of 0.08 years of life expectancy per person. Scaled up to an annual cohort this implies 10,753 patients not taking up cardiac rehabilitation due to the delay, equating to a loss of 3936 years of life expectancy. We estimate that an additional £12.3 million of National Health Service funding could be invested to alleviate the current delay.

**Conclusions:**

The current delay in many patients starting cardiac rehabilitation is causing quantifiable and avoidable harm to their long-term health; policy and research must now look at both supply and demand solutions in tackling this issue.

## Introduction

The international burden of cardiovascular disease, both on patient health and healthcare budgets, is enormous, associated with an estimated 9.43 million deaths worldwide in 2016,^1^ costing the English National Health Service (NHS) £7 billion a year to treat,^2^ and the global economy an estimated $900 billion.^3^ This burden is only expected to increase over time.^1,3^ To attempt to alleviate its impact, policy makers have sought to increase preventative activities,^4^ in addition to limiting the individual burden for patients who have cardiovascular disease.^5^ A key focus of the latter has been the drive to offer cardiac rehabilitation (CR) to eligible patients who have been diagnosed with cardiovascular disease, in an attempt to reduce the risk of future cardiac events, through a comprehensive health behaviour approach including exercise training, education (e.g. diet and physical activity promotion) and psycho-social support.

Recent research has demonstrated that CR is both highly effective^6^ and cost-effective^7^ for coronary heart disease (CHD) patients. However, despite extensive guidance on the timeframe within which CR should be started after myocardial infarction (MI) or revascularisation,^5,8^ there is significant variation in the timeliness of initiation.^9^ International research has identified that a delay in the start of CR has contributed to the poor levels of engagement with the service, both uptake^10–12^ and completion,^10,11,13^ as well as impacting the propensity to benefit from the programme.^14,15^ Previous authors have identified that this delay is the result of both patient and service-level factors.^16^ However, to date there has been no attempt to combine these factors to determine the impact of delayed start on long-term patient health and cost burden of continued cardiovascular disease on the healthcare system.

In this paper we report de-novo regression analyses exploring the impact of a delay on uptake and completion of CR using the British Heart Foundation (BHF) National Audit of Cardiac Rehabilitation (NACR) database.^17^ These regressions are used to extend an existing mathematical model of the long-term health and resource use implications of CR^7^ in order to estimate the impact of the existing delay in CR initiation in an English setting. We consider: (a) the detrimental impact of the delay on the benefits of CR; (b) the population health and cost implications of the delay; and (c) the funding that can be justified to increase the offer of timely CR.

## Methods

### What is the scale of delayed CR initiation?

**Figure 1.**
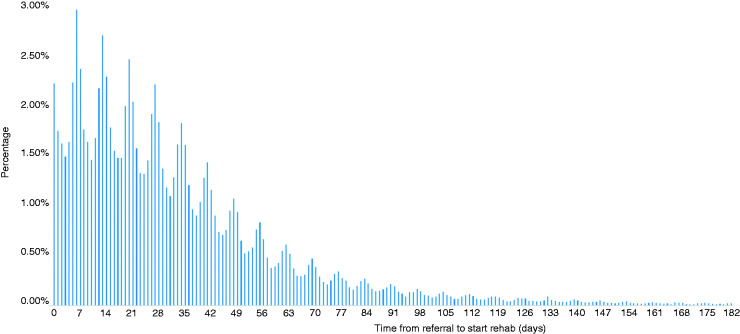
Histogram of waiting times from referral to initiation, 2015–2019.^17^

To consider the impact of a delay in CR initiation on outcomes of interest we first define what constitutes ‘timely CR’ from ‘delayed CR’. This study uses a definition of timely being a start of CR within 28 days of referral for MI and/or percutaneous coronary intervention (PCI) and 42 days for coronary artery bypass graft (CABG) patients, this is consistent with the approach taken in the current UK audit^17^ and the literature where the delay is treated categorically.^14^ Figure 1 and Supplementary Table 1 provides a histogram and summary by intervention of the time between referral and initiation of CR from the available NACR data with a cut-off of 6 months.^17^

The figure shows a significant skew in waiting times, while the majority of patients achieved the target (56.4%), many had to wait much longer. Patients who started CR within the recommended period waited a median of 15 days from referral, with those who did not start CR in the recommended period waiting a median of 49 days, see Supplementary Table 1 in the Supplementary Appendix for more details. The data also demonstrate a significant variation in the demographic and socioeconomic make-up of the two groups, with women, non-white, less deprived and employed people being more likely to have a delayed start. The impact of these differences is further explored in the regression analyses reported below.

### What does the evidence say on the impact of delay?

#### The impact of delay on uptake

When exploring the impact of a delay in CR on the rate of uptake (i.e. non-participation) it is important to note the intrinsic challenge that in order to define the impact of wait time on uptake an estimate of the wait time between referral and initiation of CR is required in both those who do and do not take up CR. However, by definition, patients who do not take up CR cannot have a CR start date, and therefore no wait time can be estimated. As a result, a proxy for the initiation date must be used, for example the initial assessment date which typically occurs just before active CR. The initial assessment is conventionally used to assess the suitability of the patient and explain the programme to them, and as such it is not part of the active intervention but intrinsically linked.

To estimate the impact of the delay on the rate of uptake, taking account of the known cofounders,^18^ we conducted a logistic regression using data routinely collected through the NACR.^17^ The regression estimates the impact of characteristics, including a categorical wait time variable, on the probability of uptake, therefore estimating the impact of the delay on non-participation in CR. The method of regression was backward stepwise, with an inclusion criteria of 0.1 and significance set at 0.05. This allowed the regression model to be adapted to include only statistically influential variables. As the quality of data reporting in routine datasets is relatively poor, for a robust analysis of uptake, a reduced cut of the NACR population was used to include four large programmes in which the data quality was known to be high. Data over a 4 year period (2016–2019) were used to inform the regression, resulting in a sample size of 2779 patients.

#### The impact of delay on completion

The second effect of a delay in CR initiation modelled in the base case analysis is the expected reduced rate of completion. Patients are most amenable to change and intervention engagement soon after a significant health shock such as CHD; therefore, their level of engagement is reduced if CR is offered with a delay. As a result, patients may still start the programme but the delay impacts their likelihood of completing it.

As with the uptake analysis we conducted a logistic regression of the NACR data, seeking to estimate the impact of the delay on completion, adjusting for known confounders. However, as data completeness and quality are much higher in the dataset for completion we were able to use the full NACR population who had started the core CR programme and a wait time recorded, again over a 4 year time period, a total of 71,423 patients.

### The mathematical model

The regression analyses conducted on the NACR dataset summarised above are carried forward to the mathematical model. By applying the results to the observed wait time and patient characteristics in the delayed CR initiation group, it is possible to estimate the expected increase in uptake and completion that could be achieved if all patients who are currently being delayed were to start CR within the recommended wait time. The parametric uncertainty associated with the regression analyses is incorporated into the health economic analysis using Cholesky decomposition to account for the correlation of the coefficients.^19^

To ensure consistency with existing research and UK policy recommendations, this analysis is constructed around an existing peer-reviewed mathematical model of the impact of CR, which was used to inform the NHS Long Term Plan^2^ and latest British Heart Foundation (BHF) strategy.^9^ Details of the model are published elsewhere,^7^ but in brief the model explores the cost-effectiveness of CR for CHD patients who are eligible for CR, including all MI and revascularisation patients using the findings of the 2016 Cochrane review of CR for CHD.^6^ The analysis concluded that CR was a cost-effective use of limited NHS resources, as while it entailed an additional cost over the lifetime of the patient (£714) it also entailed significant expected increases in patient health (0.30 quality-adjusted life-years; QALYs). This implied a cost per QALY incremental cost-effectiveness ratio (ICER) of £2395/QALY, far below the conventionally applied threshold for cost-effectiveness of £20,000/QALY.

## Results

The results of this analysis are structured to quantify the combined impact of the delay on uptake and completion, and the implications of this on the long-term patient health and cost to the healthcare provider. All of the results are presented in terms of the expected benefits that could be achieved if those patients who did not start CR within the target time did so, with those who received timely CR assumed to receive the benefits as defined by the Cochrane review^6^ and the original health economic model.^7^

### What is the combined impact of delayed CR?

The results of the regression are given in Table 1, showing that for patients with a wait time that complied with the national guidance, both uptake and completion was significantly greater than for those who had a longer wait time for CR. This implies odds ratios of 1.782 for uptake and 1.106 for completion, both at *P* values of 0.001 or less.

**Table 1.** Regression analysis of factors effecting completion rates using NACR 2015 to 2019.

Variable	Uptake			Completion		
	Coefficient	S.E.	Sig.	Coefficient	S.E.	Sig.
Gender (effect of being female)	Not significant			–0.137	0.036	0.000
Age (effect of increasing by 1 year)	–0.026	0.011	0.000	0.011	0.001	0.000
Waiting time (effect of having shorter wait time <28/42 days)	0.578	0.100	0.000	0.100	0.029	0.001
Employment (effect of being employed/retired)	–0.901	–0.227	0.000	–0.227	0.040	0.000
Ethnicity (Non-white)	0.892	0.228	0.000	Not significant		
Marital status (effect of being partnered)	1.148	0.127	0.000	0.233	0.034	0.000
Patient type (base state PCI)	Not significant					0.000
Patient type (Being CABG compared with PCI)				0.256	0.039	0.000
Patient type (being other compared with PCI)				–0.037	0.057	0.510
IMD (Base state highest deprived quintile)	Not significant					0.000
IMD (effect of being 2nd quintile)				0.166	0.049	0.001
IMD (effect of being 3rd quintile)				0.345	0.049	0.000
IMD (effect of being 4th quintile)				0.467	0.049	0.000
IMD (effect of being 5th quintile)				0.571	0.048	0.000
Constant	1.144	0.394	0.004	0.128	0.098	0.190

NACR: National Audit of Cardiac Rehabilitation; PCI: percutaneous coronary intervention; IMD: Index of Multiple Deprivation.

Applying the known patient characteristics to the results of the logistic regressions allows us to estimate the rate of uptake and completion for the group in which the CR is delayed and how they would change if CR started within the recommended wait time. These are reported in Table 2, showing that if the patients who currently received delayed CR were given it in a timely manner they would be expected to increase their uptake by 14.3% and their completion rate by 1.9%. Nationally, this implies 10,753 more patients would take up CR if the delay was removed, and 8757 more would complete the programme.

**Table 2.** Estimate of the delay on uptake and completion, and a shift to timely initiation.

	Delayed CR offer	Timely CR offer	Difference (95% CI)
Uptake	45.5%	73.4%	14.3% (7.9% to 20.4%)
Completion	59.8%	75.4%	1.9% (0.8% to 3.0%)
Combined	33.4%	45.1%	11.7% (6.9% to 16.2%)

CR: cardiac rehabilitation; CI: confidence interval.

Also of note, the positive 95% confidence intervals indicate that the delay is never expected to result in a detrimental impact on uptake or completion. This is the result of the statistical significance of the effects identified in the previous section and has important implications regarding the overall uncertainty of the conclusions drawn below.

### What is the impact of the delay on patient health and healthcare expenditure?

The impact in terms of expected patient health and healthcare costs, when these findings are applied to the baseline model, are reported in Table 3.

**Table 3.** Impact of removing the delay on average health and NHS costs per patient referred for CR.

	Costs (undisc.)	Cost (disc.)	LYs (undisc.)	QALYs (undisc.)	QALYs (disc.)*
Delayed CR offer	£8763	£7203	7.433	5.39	4.51
Timely CR offer	£8883	£7310	7.516	5.45	4.55
Difference	£120	£107	0.08	0.06	0.03
(95% CI)	(£14 to £267)	(£23 to £219)	(0.02 to 0.18)	(0.02 to 0.13)	(0.01 to 0.09)

CR: cardiac rehabilitation; disc.: values discounted at a rate of 3.5% per annum in line with NICE guidance (NICE 2013); undisc.: no discounting applied; Lys: life years; QALYs: quality-adjusted life-years.

They show that a shift from delayed to timely CR would be expected to result in an additional 0.08 life-years on average per person referred for CR (approximately one month). This results in a gain of 0.06 QALYs, 0.03 QALYs when discounted to the present value. The result is driven by more patients achieving the health gain from completing CR (0.30 QALYs).^7^ The larger proportion of the cohort receiving CR implies a greater average lifetime cost of £120, or £107 when discounted. When the cost of the higher rate of CR is excluded the difference in lifetime cost is small at £13 per person. This implies that while providing CR earlier to this group is not cost saving due to the additional CR provision, it is associated with an increase in long-term patient health at an incremental cost-effectiveness ratio of £3286/QALY.

Combining the population estimated to be currently receiving delayed CR of 34,496 (44% of the 78,997 currently receiving CR per year) and the 10,753 estimated not to take up CR as a result of the delay, gives a total population health loss due to the delay of 3936 life-years or 2792 QALYs (undiscounted) for every year when CR is not offered in keeping with national guidance. Over a 5 year timeframe this loss of patient health can be estimated as resulting in a loss of 1587 year of life across the 450,000 patients who would have CR over that period.

### What additional funding can be justified to alleviate it?

Inevitably, achieving the shift to initiation within the national guidance timeframe will require additional funding. By applying an estimate of the marginal productivity of the NHS of £12,936/QALY^20^ it is possible to calculate what NHS expenditure could be justified to achieve timely CR for all patients. This implies that an additional £315 could be justified per patient in the delayed CR group while maintaining the cost-effectiveness of the service, or £137 per patient starting CR when spread across all patients, £12.3 million across the full CR population per year. Adding this to the modelled cost of CR (£748)^7^ implies that a cost of up to £885 for CR could be justified as cost-effective should all patients receive it in line with national guidance on waiting times.

## Discussion

There is large variation in the time at which CR is delivered in the UK and internationally,^17,21^ and there is now extensive evidence that this delay is contributing to poorer uptake and completion rates, and is likely to result in decreased effectiveness of the programme. We have estimated that the delay in England is causing 3936 lost years of life across the patients’ lifetime for each year the delay endures. This analysis has also demonstrated that once the additional CR enrolments are paid for the move to earlier initiation for all patients is cost neutral, and that an additional £137 could be spent per CR patient to ensure the timely start for all, increasing the recommended cost of CR to £885.

The strength of the study is that it is the first to quantify the impact of the delay in CR initiation on uptake and completion, and to estimate the additional funding that can be allocated to alleviate it. By building on an existing peer-reviewed model, which has informed policy, this analysis ensures a consistent narrative on the latest policy facing research.

There are, however, several weaknesses associated with this analysis in addition to those in the baseline model.^7^ Firstly, in order to conduct a regression analysis for the impact of the delay on CR uptake we needed to use a proxy to estimate the wait time as well as relying on a reduced set of NACR data. There is the risk that such a proxy misses a proportion of patients who, due to a long wait for the assessment date, chose to not attend it, and thus cannot have a wait time estimated. Therefore, any estimate of the impact of delay on uptake is likely to underestimate the scale of patient failure to uptake; however, the use of such a proxy is both unavoidable and has precedent in the literature.^10–12,18^ A further limitation is that the reduced dataset may not be representative of the wider CR population, as it contained slightly more women than the full population, but not at significant levels and the average age and ethnic mix was similar. In addition, there are potential confounders such as frailty, comorbidities and rurality, which may be important differences in the timely and delayed populations, but which are not reflected in the dataset available to us and thus not the regressions conducted.

Other authors have published estimates of the impact of the delay from referral to initiation of CR on uptake and completion of the programme. Russell et al.^12^ conducted a retrospective regression analysis of 599 patients referred to a single centre CR programme in Canada, concluding an odds ratio of 0.99 (95% confidence interval of 0.98 to 0.99) for an additional wait of one day on uptake. Although the nature of the regression makes direct comparison with our analysis difficult, we consider the result to be comparable. Similarly, considering the impact of a delay on completion, Marzolini et al.^13^ conducted a regression analysis which incorporated a consideration of delay on completion, in a large dataset of CABG patients in Canada between 1995 and 2012. The authors similarly found a statistically significant correlation between log wait time and non-completion (coefficient of 2.215, *P* < 0.001). Marzolini et al. additionally explored the impact of delays in the referral to CR, an element which is not included in this analysis as it refers to a different policy question regarding the speed of referrals, and the health threshold at which patients become eligible for CR, rather than failures of the programmes to achieve timely start targets.

An additional weakness is that while we have been able to conduct an exploratory analysis to estimate the additional impact of incorporating the role of a delay in initiation on CR outcomes, reported in the Supplementary Appendix, the informative estimates are highly uncertain. Inevitably, the analysis indicates that if the impact of the delay on outcomes were incorporated the loss of patient health as a result would be even worse than in the current model, suggesting our analysis underestimates the benefits of timely CR. Further research and data collection are needed to understand the factors that influence different CR outcomes, such as long-term physical fitness.

We recommend that future studies explore the key policies and interventions that may effectively alleviate the delay, specifically further exploring whether it is a supply or demand side issue.^16^ In addition, further routine data collection is require on the reasons patients do not engage with CR programmes, and the long-term impact of factors such as wait time on the effectiveness of the programme.

## Supplemental Material

CPR912625 Supplemental Material - Supplemental material for Quantifying the impact of delayed delivery of cardiac rehabilitation on patients’ health Click here for additional data file. Supplemental material, CPR912625 Supplemental Material for Quantifying the impact of delayed delivery of cardiac rehabilitation on patients’ health by Sebastian Hinde, Alexander Harrison, Laura Bojke and Patrick Doherty in European Journal of Preventive Cardiology
