# Supplementary material for: Quantifying the impact of delayed delivery of cardiac rehabilitation on patients’ health
Source: Eur J Prev Cardiol. 2020 Mar 25;27(16):1775–81. doi: 10.1177/2047487320912625 (PMC7564289; doi:10.1177/2047487320912625)
Supplement: CPR912625 Supplemental Material - Supplemental material for Quantifying the impact of delayed delivery of cardiac rehabilitation on patients’ health [file CPR912625_Supplemental_Material.pdf]

Supplementary appendix

Section 1) Summary statistics of waiting time by patient type

Table A1: summary statistics of waiting time by patient type

Characteristic		Timely start		Delayed start		Total	
		Mean	SD	Mean	SD	Mean	SD
Age (years)		64	11	63	12	64	11
Treatment category (days)	MI	13.1	8.2	57.4	29.4	31.6	29.6
	MI and PCI	14.2	8.0	54.4	27.4	30.6	27.1
	PCI	14.7	7.6	56.4	29.0	31.3	28.0
	CABG	22.7	12.7	70.7	28.1	42.2	31.2
	Total	15.8	9.6	58.4	28.9	33.2	28.8
		Count	%	Count	%	Count	%
Age Grouped	<40	866	2.0%	567	2.5%	1433	2.1%
	41-50	4804	10.9%	2717	11.8%	7521	11.2%
	51-60	11540	26.3%	6063	26.3%	17603	26.3%
	61-70	13538	30.8%	6896	29.9%	20434	30.5%
	71-80	10299	23.5%	5201	22.5%	15500	23.1%
	81+	2843	6.5%	1652	7.2%	4495	6.7%
Gender	Male	34319	78.8%	17209	75.0%	51528	77.5%
	Female	9243	21.2%	5731	25.0%	14974	22.5%
Ethnicity	White	33237	75.7%	16812	72.8%	50049	74.7%
	Non-white	10653	24.3%	6284	27.2%	16937	25.3%
Employment	Employed/ Retired	23641	82.0%	13258	84.8%	36899	83.0%
	Unemployed	5199	18.0%	2368	15.2%	7567	17.0%
Marital Status	Single	6708	22.1%	3883	24.1%	10591	22.8%
	Partnered	23712	77.9%	12243	75.9%	35955	77.2%

Section 2) Additional impact of delay on outcome scenario

A number of studies have explored the impact of the delay on the effectiveness of CR. Johnson et al.[1] conducted multivariate analysis on a single centre US dataset, finding delay was significantly correlated with peak exercise capacity during the programme. Similarly, Fell et al. [2] and Sumner et al. [3], conducted regression analysis on the NACR dataset, finding the delay was correlated with a range of factors: physical activity, physical fitness, incremental shuttle walk test score, anxiety, and depressive symptoms.

While the repeated demonstration of an effect of the delay on short term physical and mental outcomes suggests an effect above and beyond the poorer level of uptake and completion, the lack of long-term analyses using outcomes directly relevant to the baseline model makes the incorporation of these studies challenging and necessitating on significant assumptions. For this reason the inclusion of the impact of delays on outcomes was not included in the primary analysis in this study, but a scenario was constructed which is presented in this appendix.

In order to incorporate the findings of these studies for a scenario analysis into the model it was first necessary to find a study which correlated any of the outcomes in these three studies to the outcomes relevant to the baseline model. Only a single outcome was identified which achieved this requirement, linking the estimated difference in self-reported physical activity from Fell et al. [2] to all cause and cardiovascular related mortality through the study by Mok et al. [4].

Fell et al. [2] report physical activity in terms of the proportion of patients achieving the recommended 150 minutes of moderate activity per week, finding an OR of 0.863 per additional day of delay. Mok et al. [4] identified, through interrogation of a population cohort in the UK, that an increase in physical activity of 1kJ/kg/day, assumed by them to be equivalent to being inactive at baseline to achieving the 150 minute target after 5 years, was associated with hazard ratios (HRs) of 0.71 for cardiovascular mortality, and 0.76 for all cause. Clearly, the combining of these studies to link the findings of Fell to our baseline model variables requires a number of significant assumptions regarding the duration of the observed effect and the comparability of the physical activity outcomes used in the two studies. If we assume that the outcome observed in Fell is life-long, the outcomes perfectly equivalent, and that the HR reported for cardiovascular mortality applied to all of the outcomes which patients are modelled as benefitting from CR it is possible to combine the outcomes to determine that the delay has a detrimental effect of 1.017 on all outcomes. This implies that the delay in CR provisions makes CR less effective in all patients by a factor of 1.017, so a relatively small impact relative to the overall benefit of CR.

The impact of this additional effect when added to the result reported in the main paper are reported in Table A2.

Table A2: model results from addition of delay impact on outcomes

	Costs (undisc.)	Cost (disc.)*	LYs (undisc.)	QALYs (undisc.)	QALYs (disc.)*
Delayed CR offer	£9,252	£7,565	7.79	5.64	4.73
Timely CR offer	£9,389	£7,629	7.91	5.70	4.80
Difference	£137	£65	0.12	0.05	0.06

While we consider the inclusion of the impact of delay on CR effectiveness to be too uncertain to include as the primary analysis it is important to note that Fell demonstrates a statistically significant impact of the delay on a number of short term outcomes and that offering CR in a timely way will, within reason, always be expected to only improve effectiveness. This implies that we would expect the benefits of timely CR to be greater than those stated in the primary analysis, and thus the justifiable expenditure to increase timeliness greater than is reported.

1. Johnson DA, Sacrinty MT, Gomadam PS, et al. Effect of early enrollment on outcomes in cardiac rehabilitation. *The American journal of cardiology* 2014; 114: 1908-1911. 2014/12/03. DOI: 10.1016/j.amjcard.2014.09.036.
2. Fell J, Dale V and Doherty P. Does the timing of cardiac rehabilitation impact fitness outcomes? An observational analysis. *Open Heart* 2016; 3: e000369. 2016/02/13. DOI: 10.1136/openhrt-2015-000369.
3. Sumner J, Bohnke JR and Doherty P. Does service timing matter for psychological outcomes in cardiac rehabilitation? Insights from the National Audit of Cardiac Rehabilitation. *European journal of preventive cardiology* 2018; 25: 19-28. 2017/11/10. DOI: 10.1177/2047487317740951.
4. Mok A, Khaw K-T, Luben R, et al. Physical activity trajectories and mortality: population based cohort study. 2019; 365: l2323. DOI: 10.1136/bmj.l2323 %J BMJ.
